# THE IMPACT OF AN INTENSIVE LIFESTYLE INTERVENTION IN TYPE 2 DIABETES ON LONG-TERM MULTIMORBIDITY

**DOI:** 10.1093/geroni/igae098.0389

**Published:** 2024-12-31

**Authors:** Ian Davis, Peter Huckfeldt, Mark Woodhouse, Mark Espeland, Lynne Wagenknecht, Dana Goldman, Marcel Salive, Ann Harada

**Affiliations:** University of Southern California, Los Angeles, California, United States; University of Minnesota School of Public Health, Minneapolis, Minnesota, United States; University of Minnesota, Minneapolis, Minnesota, United States; Wake Forest University School of Medicine, Winston-Salem, North Carolina, United States; Wake Forest University School of Medicine, Winston-Salem, North Carolina, United States; University of Southern California, Los Angeles, California, United States; National Institute on Aging, Baltimore, Maryland, United States; Leonard D. Schaeffer Center for Health Policy & Economics, Los Angeles, California, United States

## Abstract

Multimorbidity, or the accumulation of multiple chronic diseases, is an important clinical issue in aging and is associated with geriatric syndromes, physical and cognitive function declines, increased health care costs, and poorer health-related quality of life. This study aims to better understand multimorbidity occurrence and accumulation over the postintervention period (2013-2019) of Look AHEAD, which randomized 5145 individuals aged 45-76 in 2001-2004 with type 2 diabetes and overweight/obesity to an intensive lifestyle intervention (ILI) or a control arm receiving diabetes support and education (DSE). The ILI was previously found to reduce weight, improve diabetes control, and reduce diabetes complications. The ILI was also associated with relative improvements in self-reported multimorbidity during the intervention period. We included 2940 participants consenting to linkage of their trial data with Medicare data, focusing on participant years with Medicare fee-for-service coverage during the postintervention period. We identified conditions from the list developed by the “Department of Health and Human Services Initiative on 21 Multiple Chronic Conditions (MCC)”. We estimated ILI versus control differences in numbers of conditions using linear models that control for baseline demographic and clinical characteristics and study site. We found similar levels of multimorbidity for the ILI and DSE groups (4.21 versus 4.31, p=0.41). This result suggests that the positive impacts of the ILI on diabetes control did not translate to long-term reductions in multimorbidity.

